# Recent Nanotherapeutic Advancements Against HIV-Associated Neurocognitive Disorders (HAND)

**DOI:** 10.3390/biom16050728

**Published:** 2026-05-15

**Authors:** Riddhi Trivedi, Avinash Gothwal, Buddhadev Layek, Jagdish Singh

**Affiliations:** Department of Pharmaceutical Sciences, North Dakota State University, Fargo, ND 58102, USA

**Keywords:** HAND, blood–brain barrier, antiretroviral therapy

## Abstract

HIV-associated neurocognitive disorders (HAND) arise from HIV infection of the central nervous system, resulting in chronic neuroinflammation and progressive neuronal damage that impair cognitive, motor, and behavioral functions. Clinically, HAND encompasses a spectrum of neurological impairments ranging from asymptomatic neurocognitive impairment to severe HIV-associated dementia. Despite the widespread use of combination antiretroviral therapy (cART) and significant improvements in the life expectancy of people living with HIV, HAND remains prevalent and continues to pose a major clinical challenge. One of the primary limitations of cART is the limited penetration of many antiretroviral drugs across the blood–brain barrier (BBB), thereby allowing the persistence of viral reservoirs within the CNS and contributing to sustained neuroinflammation and neuronal damage. To address these challenges, novel nanotherapeutic strategies have been developed to enhance the delivery of antiretroviral agents to the brain. These approaches include targeted delivery systems and the co-delivery of therapeutics across the BBB through mechanisms such as receptor-mediated transcytosis and other transport pathways. In this review, we discuss the pathophysiological challenges associated with HAND and recent advances in nanotherapeutic approaches designed to improve treatment efficacy. We also discuss the current state of the art in vitro and in vivo models used to test the efficacy of these advanced therapeutics. Finally, we outline the remaining challenges and future prospects for the development of nanotherapeutics to improve the treatment of HAND.

## 1. Introduction

Human immunodeficiency virus (HIV) infection affects millions of people worldwide and remains a significant global health challenge. Currently, more than 34 million people are infected with HIV, and adding to the toll as many as 1.7 million new cases every year [1]. According to the reports, 39–70% of people infected with HIV suffer from some form of neurocognitive impairment [2]. The virus can reach the central nervous system (CNS), causing damage to the brain and spinal cord, which may lead to cognitive decline, motor dysfunction, and behavioral abnormalities, collectively known as HIV-associated neurocognitive disorders (HAND). HAND encompasses a range of cognitive deficits, ranging from mild impairments to severe dementia, and significantly impacts the quality of life and long-term prognosis of affected individuals [3]. Based on their severity, HAND is classified into three distinct categories: Asymptomatic Neurocognitive Impairment (ANI), Mild Neurocognitive Disorder (MND), and HIV-Associated Dementia (HAD). This classification relies on neuropsychological testing and additional clinical assessments of memory, attention, executive function, motor skills, and language. As per the ICD-11 (6D85.3), “dementia develops during the course of confirmed HIV disease, in the absence of a concurrent illness or condition other than HIV infection that could explain the clinical features”. The course of dementia due to HIV varies, including resolution of symptoms, a gradual decline in functioning, improvement, or fluctuation in symptoms [4].

An evaluation of daily functioning further highlights the impact of cognitive decline on everyday activities. Diagnostic techniques such as neuroimaging and cerebrospinal fluid (CSF) analyses provide additional information regarding HAND-associated neuroinflammation in the CNS. Individuals with ANI typically maintain daily functioning but may experience subtle difficulties with attention, memory, or executive function. In contrast, MND is characterized by more pronounced cognitive decline, with moderate impairments in concentration, executive function, and other cognitive domains. HAD is the most severe form of HAND, with individuals exhibiting significant cognitive decline and marked impairment in daily functioning [5,6]. HAND is more prevalent in women due to a complex interplay of psychological, biological, and clinical factors. Additionally, age and depressive symptoms also increase neurocognitive risk in women [7].

The underlying mechanism of HAND progression remains unclear. HIV is believed to enter the CNS via infected macrophages and lymphocytes and to persist mostly in perivascular macrophages and microglia [8]. Recent studies have identified single-nucleotide polymorphisms, including *MTND4P3 [rs4718789-T]*, *RNA5SP231 [rs4718789-T]*, and *MSH6 [rs2098242-T]*, as significant contributors to HAND pathogenesis. Additionally, miRNAs such as *hsa-miR-16-5p*, *hsa-miR-320a,* and *hsa-miR-335-5p*, as well as transcription factors *THRA* and *NEUROD6*, have been implicated in HAND [9]. Several reports indicate that the degree of cognitive impairment in patients with HAND is directly related to CSF viral load [10,11,12]. Once HIV reaches the brain, it infects macrophages and microglia, leading to neuronal apoptosis. Activation of these cells is central to HAND pathogenesis [13]. Toxin production subsequently causes astrocytic and neuronal dysfunction [14]. Glial cell activation is a hallmark of many neurodegenerative and neuroinflammatory disorders. In people living with HIV, cognitive impairment is characterized by neuronal loss and neuroinflammation, even among those receiving long-term antiretroviral therapy [13,15,16].

Inflammatory cytokines also cause neuronal damage, which is mediated by alterations in the glutamate pathway. Different viral proteins, such as the transcriptional transactivator (Tat), viral protein R (Vpr), negative regulatory factor (nef), and glycoprotein 120 (gp120), have been extensively studied for their roles in neuropathogenesis [17,18,19]. The Tat protein has been linked to NMDA receptor-mediated calcium signaling, which triggers programmed cell death in primary human neurons [14]. Both Tat and nef trigger macrophages to release quinolinic acid, a known neurotoxin [20]. gp120 can interact with neuronal receptors and contribute to neuronal injury. It also dysregulates glutamate uptake in astrocytes. As a result, extracellular glutamate levels rise, leading to excitotoxicity and, eventually, neuronal death [20,21,22]. The viral protein Vpr induces neuronal death by disrupting mitochondrial functions in the cell [13].

Current treatment strategies primarily rely on cART, but the restricted permeability of the blood–brain barrier (BBB) limits the therapeutic potential of these drugs against HAND. However, nanocarrier-mediated drug-delivery approaches have been investigated as a new treatment for HAND. This review discusses the current challenges and prospects of nanotherapeutics for the treatment of HAND. Moreover, the current state of the art of in vitro and in vivo models used to test the efficacy of these advanced therapeutics has also been included.

## 2. Challenges in Current Treatment Approaches

Since the introduction of cART in 1997, HIV-related morbidity has significantly decreased. Antiretroviral drugs are categorized based on the stage of the HIV life cycle they inhibit. Although cART effectively suppresses systemic viral replication, many antiretroviral agents have limited ability to cross the BBB. As a result, managing HAND remains a persistent challenge [23]. The BBB is a selectively permeable physiological barrier that preserves CNS homeostasis by tightly regulating the passage of substances from the bloodstream into the brain.

Drug molecules smaller than ~400 Da can cross the BBB primarily via lipid-mediated diffusion. Some small molecules may also enter the brain via alternative mechanisms, but most are actively removed by efflux transporters such as P-glycoprotein (P-gp) and multidrug resistance proteins [24]. Antiretroviral (ARV) drugs can further induce P-gp expression, which limits their therapeutic concentrations in the brain [25]. This limited availability of these drugs in the brain cannot restrict the viral reservoir in the CNS [26].

Latent or hidden HIV reservoirs present a major challenge to achieving a functional cure for the disease. Following treatment interruption, the virus from these reservoirs can re-enter the circulation, causing a rapid viral rebound in plasma within weeks. Macrophages and microglial cells act as key cellular reservoirs and can re-trigger infection in peripheral tissues after treatment discontinuation [27]. Infected macrophages significantly affect the proteome of cerebral endothelial cells, resulting in damage to the BBB and facilitating the entry of HIV and circulating neurotoxins into the CNS [28]. Similarly, astrocytes also act as HIV reservoirs under specific conditions, such as priming by IFN-ϒ [29]. Overall, limited drug permeability across the BBB remains a fundamental obstacle to developing effective therapeutic strategies for eliminating HIV reservoirs in the CNS [30].

Moreover, the efflux transporters pump out drugs along with the harmful substances and limit the effectiveness of drugs that cross the BBB [22]. Metabolic barriers also play a significant role in limiting drug concentrations through enzymatic degradation before drugs reach the target site [31]. Highly lipophilic drugs may cross the BBB via passive diffusion but often lack target specificity due to their uptake mechanism [32]. This results in non-targeted dispersion in the brain, rather than delivery to the target site. Lack of specificity of the CNS disease-treating drugs can expose other healthy organs and can cause neurotoxicity, excitotoxicity, inflammation, and cell death [32,33,34]. This lack of precision is also a major challenge in achieving an effective treatment regimen.

## 3. Nanotherapeutics for HAND

Concerns about the failure of cART in HAND patients remain a global challenge. In recent years, nanocarrier-based drug delivery systems have demonstrated immense potential to overcome BBB-related challenges and improve drug pharmacokinetics in the brain. Additionally, nanoparticles enable site-specific or targeted drug delivery, which can diminish drug-associated toxicity and enhance patient compliance [35].

**Figure 1:**
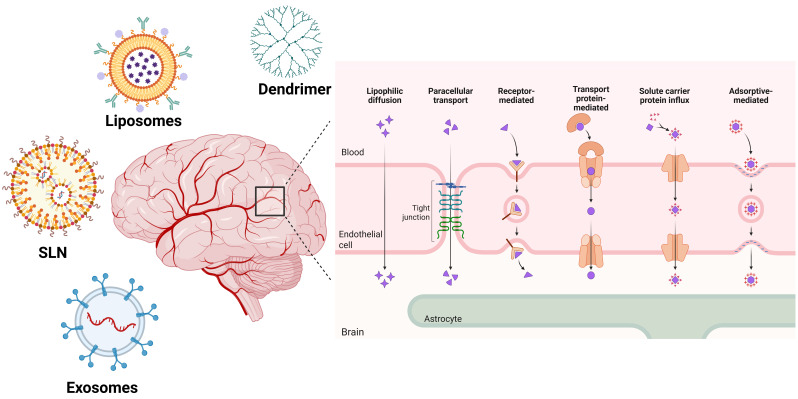
Schematic representation of different nanocarriers and their mechanisms of transcytosis across the blood–brain barrier (BBB). SLN: solid lipid nanocarrier. The figure was created using BioRender (Created in Biorender by Jagdish Singh (2026): https://app.biorender.com/).

Nanocarriers and drugs are internalized via different transcytosis mechanisms; for example, lipid nanocarriers and lipid-soluble small molecules can cross the BBB via lipophilic diffusion (Figure 1). Paracellular transport is restricted for large molecules and becomes more permeable under pathological conditions. Functionalization of the nanocarriers with targeting ligands (CPPs, small receptor-binding effector molecules) exploits the receptor-mediated transcytosis pathway for internalization. Solute-carrier protein influx also functions similarly to receptor-mediated transcytosis but operates against the concentration gradient. Neurotransmitters and drugs like L-DOPA often follow this pathway, whereas adsorptive-mediated transport is based on electrostatic interactions between the drug/nanocarrier and the cell membrane [36].

A variety of nanocarrier systems have been developed to effectively deliver drugs to the CNS. Their engineered physicochemical properties allow for the incorporation of targeting ligands that guide therapeutic cargo to specific sites of action. In the following section, we discuss nanoparticles that have been explored for drug delivery in the treatment of HAND.

### 3.1. Lipid Nanocarriers

Several lipid-based nanocarriers have been studied for the delivery of ARV drugs to the CNS, including liposomes, solid lipid nanoparticles (SLN), and nanostructured lipid carriers (NLC). Liposomes are biodegradable phospholipid bilayer vesicles that can encapsulate hydrophilic and hydrophobic drugs, making them suitable for CNS drug delivery [35]. A liposomal formulation encapsulating the antiviral drug foscarnet exhibited a 13-fold higher brain uptake than the free-drug treatment group [37]. The systemic clearance of the liposomal drug was 77-fold lower than that of the free drug.

The antiviral activity and bone marrow toxicity of 3′-azido-3′-deoxythymidine (AZT) encapsulated in liposomes were evaluated in *C57BL/6* mice [38]. The liposomal formulation enhanced AZT distribution to the liver, spleen, and lungs while reducing its accumulation in the bone marrow. AZT exhibits bone marrow toxicity at >2 mg/kg/day after five administrations, whereas the liposomal formulation showed no detectable toxicity across doses ranging from 0.08 to 10 mg/kg/day. In another study, nevirapine (NVP)-loaded liposomes were evaluated for drug loading efficiency, release kinetics, and BBB penetration. The findings indicated that this liposomal delivery system enabled efficient drug delivery while reducing systemic toxic effects, highlighting its potential to improve antiretroviral therapy [39].

Novel magneto-liposomes were developed as multi-component sustained-release formulations containing the antiretroviral drugs tenofovir and nelfinavir, the latency-reactivating agent disulfiram, and methamphetamine, a drug of abuse that accelerates HIV pathogenesis [40]. Results showed that these components were released from the liposomes for up to 10 days and reduced in vitro HIV-1 infection by 40–50%. Furthermore, transport across the BBB increased to approximately 15% following magnetic treatment (0.8 T). In a separate study, cannabidiol-encapsulated liposomes demonstrated reduced p24 levels and long-term HIV gene expression, increased APO levels, and attenuated mitochondrial reactive oxygen species production in infected microglia [41].

SLNs represent a relatively new class of lipid-based nanocarriers. These spherical particles are composed of biodegradable, biocompatible solid lipids with melting points above 37 °C, thereby allowing them to remain solid during administration. Their hydrophobic nature and small size enable SLNs to efficiently cross the BBB and evade P-gp efflux transporters.

This capability is further enhanced when SLNs are surface-functionalized with monoclonal antibodies, poloxamer 407 (P407), and polysorbate 80 (Tween 80) [42]. Physicochemical and cellular uptake studies showed that lipid composition influenced particle size and zeta potential, while the P407/Tween 80 coating stabilized the SLNs and reduced their uptake by macrophages. Furthermore, the presence of Tween 80 and antibody grafting improved endothelial permeability and facilitated targeted delivery of saquinavir across the BBB.

Apolipoprotein E–functionalized SLNs exhibited reduced cytotoxicity toward the human cerebral microvascular endothelial cell line (hCMEC/D3) and showed enhanced permeability across the hCMEC/D3 monolayer, with approximately a 1.5-fold greater transport compared with unfunctionalized SLNs [43]. Similarly, SLNs loaded with the azapeptide inhibitor of HIV-1 protease, atazanavir, exhibited no detectable toxicity in hCMEC/D3 cells and showed increased cellular accumulation within the endothelial cell monolayer, indicating improved uptake and potential transport across the BBB [44]. In another study, researchers assessed the permeability of three antiretroviral drugs (stavudine, delavirdine, and saquinavir) encapsulated in SLNs across the BBB. These SLN formulations achieved 3- to 16-fold greater BBB permeability than the free drugs [45]. Furthermore, ritonavir-loaded SLNs significantly inhibited HIV-1 activity in vitro, further supporting the utility of SLN-based nanocarriers for improving the delivery and therapeutic efficacy of antiretroviral drugs in the brain [46].

NLCs are also extensively studied for targeted drug delivery. Composed of a mixture of solid and liquid lipids, NLCs offer several advantages, including the prevention of drug aggregation, immobilization of the encapsulated drugs, biodegradability, controlled drug release, and preparation without organic solvents [47,48]. These features make NLCs promising drug-delivery carriers for the treatment of HAND. For instance, atazanavir is a poorly bioavailable drug that limits its therapeutic efficacy against HAND. Encapsulation in NLCs demonstrated a 2.75-fold higher C_max_ and a 4-fold higher bioavailability in the brain compared to the free drug [49].

Similarly, NLC-based delivery of etravirine improved its brain pharmacokinetics, and the developed formulation acted as a multi-site targeted therapy for eradicating viral loads from several anatomical reservoirs [50]. In another study, intranasally administered tenofovir disoproxil fumarate-loaded NLCs significantly improved pharmacokinetic parameters, including prolonged mean retention time and a higher C_max,_ compared to the free drug [51]. The scarcity of studies investigating lipid-based nanocarriers for HAND highlights an important gap in fully utilizing their potential for developing effective therapeutics.

### 3.2. Polymeric Nanocarriers

Polymeric nanocarriers have also been explored for targeted drug delivery to the brain to effectively manage neurological disorders. Different polymeric nanoparticles have been studied for their biodegradability, ability to encapsulate both hydrophilic and hydrophobic drugs, controlled-release properties, protection of drugs from degradation, and improved permeability across the BBB [52]. For example, poly(butyl cyanoacrylate) (PBCA) nanoparticles have been shown to increase the permeability of zidovudine and lamivudine across the in vitro BBB severalfold [53]. However, degradation of PBCA can generate harmful formaldehyde by-products; therefore, safer alternative polymers have been explored as more suitable carriers for brain-targeted drug delivery. Table 1 lists various nanocarriers used for brain-targeted delivery against HAND.

Polylactide (PLA) and poly(lactide-co-glycolide) (PLGA) are FDA-approved polymers and are among the most commonly used materials for biomedical applications. The degradation products of PLA and PLGA are lactic acid and glycolic acid, which enter the tricarboxylic acid cycle, where they are ultimately converted into CO_2_ and water and eliminated from the body. The tunable properties of these polymers enable easy surface modification with coating polymers, such as polyethylene glycol (PEG), and targeting ligands. Such modifications enhance systemic circulation time, reduce phagocytic uptake, and improve permeability across the BBB [56]. For instance, PLA-PEG nanoparticles improved the systemic circulation time of zidovudine by minimizing phagocytosis (Table 2) [57].

Building on these advantages, PLGA- and PLA-based nanoparticles have been widely investigated for brain-targeted delivery of antiretroviral drugs. PLGA nanoparticles loaded separately with ritonavir, lopinavir, and efavirenz demonstrated sustained drug availability in the brain for up to 28 days, whereas the corresponding free drugs were eliminated within 2 days [58]. Moreover, these nanoparticulate formulations exhibited improved in vitro antiviral efficacy by prolonging the inhibition of *HIV-1 ADA* replication. In another study, luminescent carbon dot-tagged PLGA nanoparticles loaded with darunavir enhanced the drug’s bioavailability and therapeutic efficacy [59]. The nanoparticles crossed the artificial BBB model and inhibited metalloproteinase-9, a key factor in HIV-related neurological disorders. Similarly, surface-modified PLA nanoparticles conjugated with the Tat peptide increased the bioavailability of ritonavir by nearly 800-fold compared with the free drug [64]. Collectively, these findings highlight the excellent targeting and drug-delivery potential of engineered polymeric nanocarriers for the brain, as they not only improve pharmacokinetics but also maintain therapeutic drug concentrations for extended periods, thereby enhancing treatment outcomes against CNS viral reservoirs.

### 3.3. Miscellaneous Nanocarriers

Several other nanocarrier systems have also been explored for drug or gene delivery in the treatment of HAND. For example, a carbosilane dendrimer-siRNA dendriplex demonstrated higher permeability across in vitro bovine brain microvascular endothelial cells and efficiently transfected HIV-infected human astrocytes. Importantly, the dendriplex did not exhibit toxicity toward uninfected or healthy astrocytes, and the downregulation of GAPDH and the suppression of HIV-1 replication confirmed its therapeutic efficacy [60]. In another study, a magnetic nanoparticle- and exosomal extracellular vesicle-coupled nanocarrier system carrying the *anti-HIV T20* peptide (T20) was evaluated for targeted delivery and therapeutic efficacy. The T20 peptide-loaded nanocarrier was capable of protecting neurons without compromising BBB integrity [61].

Interestingly, nanocrystals containing rilpivirine and cabotegravir were administered via microneedles on both sides of the face in rats, resulting in sustained drug bioavailability for up to 21 and 28 days, respectively [62].

## 4. Animal Models to Study HIV and Neuropathogenesis

Animal models are valuable for investigating various aspects of HIV infection that cannot be studied in humans due to ethical constraints and the considerable biological variability present in clinical populations. These models facilitate the examination of viral pathogenesis, host–virus interactions, neuroinflammation, and therapeutic interventions under controlled experimental conditions. In particular, rodent models have played a critical role in studying HAND and evaluating potential treatment strategies. Key rodent models used to study HIV infection and associated neurocognitive impairment in vivo include humanized mouse models, HIV-transgenic mice, and EcoHIV (chimeric virus) infection models [65].

### 4.1. Humanized Mouse Models

*Humanized mouse models* have been commonly used to study virus–host dynamics. These mice are developed using severe combined immunodeficiency (SCID) genetics and possess a DNA-dependent protein kinase catalytic subunit deficiency, which restricts the function of B and T cells [66]. The HIVE (HIV-1 Encephalitis) mouse model was among the first humanized models introduced to study NeuroAIDS or HAND. It was created by injecting the HIV-1-infected human monocyte-derived macrophages into the basal ganglia of immunodeficient mice [67]. This model was later used to study peripheral immunity, with the ultimate goal of improving understanding of the adaptive immune system in HIV neuropathogenesis. Non-obese diabetic mice were bred with mice carrying a *SCID* genetic background to produce mice reconstituted with human peripheral lymphocytes (huPBLs) [68,69]. These huPBL mice were injected with HIV-1-infected human monocytes. However, they exhibit high mortality and a short lifespan (four to five weeks) due to graft-versus-host disease, in which human lymphocytes recognize mouse host cells as foreign and attack them [68].

The bone marrow-liver-thymus (BLT) mouse model is a more recently developed humanized mouse model. It is generated by transplanting fragments of human fetal thymus and liver tissue into irradiated *NOD-SCID* or *NSG* mice, followed by transplantation of human CD34^+^ hematopoietic stem cells, usually isolated from the same fetal liver tissue [70]. A key feature of the *BLT* model is the development of human T cells within a human thymus, which more closely mimics clinical conditions. Additionally, studies have demonstrated that *BLT* mice infected with HIV-1 contain measurable levels of viral RNA and DNA in the brain, indicating that this model may serve as a valuable tool for exploring mechanisms and therapies for HAND [71]. A common drawback of humanized mouse models is the development of host-versus-graft disease, which limits lifespan and complicates long-term or age-related studies.

### 4.2. HIV-Transgenic Mice

Transgenic rodent models were among the first approaches to model CNS infections, as they express HIV proteins in the brain. The *gp120* transgenic mouse model, which expresses *CXCR4-tropic gp120* exclusively in astrocytes, was one of the first models used to study the role of viral proteins in the brain. These mice develop age-related (~3 months of age) memory impairments, and the model has helped identify cellular pathways involved in gp120-mediated neurotoxicity [72,73].

Similarly, a Tat-transgenic mouse model was developed in which the HIV Tat protein is expressed under a doxycycline-dependent GFAP promoter, leading to Tat-dependent pathologies such as astrogliosis, monocyte and T-cell infiltration, and premature mortality [74]. More recently, transgenic mouse models have been developed to express the Vpr protein selectively in myeloid cells of the nervous system, resulting in CNS abnormalities [75].

### 4.3. Chimeric Virus Infection Model

Chimeric virus infection models are considered safer and more robust than traditional mouse models for studying HIV infection. These models are generated by infecting wild-type mice with a chimeric virus that combines elements of ecotropic murine leukemia virus (MLV) with HIV. The chimeric virus (known as EcoHIV or EcoNDK) was created by replacing the *HIV gp120* envelope protein with MLV gp80, making the virus murine-specific [76]. The gp80 protein facilitates viral entry into target cells by cleaving into SU (gp70) and TM (p15E). The TM unit acts as a membrane anchor, while the SU subunit binds to the receptor, enabling viral entry into cells [77].

EcoHIV was created using a laboratory-derived NL4-HIV-1 strain, a molecular clone of HIV belonging to subtype clade B [78,79], whereas EcoNDK was developed from HIV subtype clade D [80]. This chimeric virus lacks gp120, which normally mediates binding to human cells, but can still infect murine monocytes, macrophages, and T cells. The infected virions disseminate throughout multiple organs and trigger antiviral immune responses [78]. The virus can also spread through sexual contact in mice, similar to HIV transmission in humans [81,82].

Chronic infection in mice leads to cognitive impairments, particularly in spatial learning and memory, resembling deficits observed in cART-treated individuals [83]. Overall, this model is well-suited for studying mechanisms of HIV infection and associated cognitive impairment due to its similarity to aspects of human HIV infection, ease of implementation, and safety. This model has been widely used to investigate HIV-associated cognitive deficits, examine the role of drug abuse in HIV replication in CNS cells [84], develop an HIV vaccine [85], and model neuroinflammation and BBB damage [86].

### 4.4. Simian-Human Immunodeficiency Virus

*Simian-human immunodeficiency viruses* (SHIVs) have been studied in non-human primate models to elucidate mechanisms of virus transmission, immunopathogenesis, and therapeutic interventions [87]. SHIVs are chimeric viruses that encode HIV-1 Env within the SIV backbone. Across species, macaque-adapted SHIV-infected Chinese- and Indian-origin rhesus macaques exhibited accelerated pathogenesis, high viral loads, and immunopathogenesis [88,89]. This model begins to develop neurodegeneration from three to six months post-infection (Table 3).

Although SHIV animal models have been widely explored and validated for testing treatment strategies, these models are less suitable for latency and cure studies. The model should demonstrate ongoing viral replication over time and persistence on ART to be used to study the effects of drugs on viral load suppression or eradication. However, not all SHIV models meet this requirement. Recently, Env375-transmitted/founder SHIVs have been attempted to be validated for the latency and efficacy studies [93].

## 5. Future Prospects

Preliminary studies using different nanocarrier platforms, including liposomes, polymeric nanoparticles, dendrimers, and exosome-mimetic vesicles, have demonstrated effective delivery and distribution within the CNS. These nanocarriers improve the pharmacokinetic profiles of ART drugs, anti-inflammatory compounds, and gene-editing payloads, while enabling controlled drug release. Surface modifications of these platforms with targeting ligands (e.g., transferrin or cell-penetrating peptides) facilitate transcytosis across the BBB, thereby increasing drug accumulation in specific brain regions. Additionally, these nanocarriers can co-deliver small molecules and nucleic acids, allowing simultaneous suppression of viral replication and attenuation of neurodegenerative pathways.

Looking forward, smart nanocarriers may be engineered to release drugs in response to pH changes, oxidative stress, or enzyme-mediated mechanisms. Such systems could provide dynamic control over drug release, therapeutic activity, and early endosomal escape. However, clinical translation of these nanocarrier-mediated therapies will require rigorous assessment of long-term safety, immunogenicity, and scalable manufacturing processes. Nonetheless, the convergence of nanotechnology and neuroHIV research represents a promising paradigm for precision therapy in HAND.

Recently, researchers found that despite the availability of cART, HAND prevalence remains high (~40–50% globally), even in virally suppressed patients. Additionally, the neuropsychological profiles of non-Western and Western cohorts largely mirror one another, and immunosuppression remains the primary risk factor across settings [94]. Similarly, in a different report, researchers evaluated the validity of cognitive screening tools for HAND identification. After analyzing 19 studies, the authors reported significant variability in criterion validity, mainly due to inconsistent explanations of impairment and demographic differences [95]. A study also claimed that the effect of HIV on cognition in women is very small (~0.05–0.09 SD units), except among women with low literacy levels and HIV-related comorbidities [96]. Therefore, universal data harmonization, longitudinal studies, and comparative studies between well-matched groups should be conducted to better understand asymptomatic impairment and to identify more reliable biomarkers [94,96].

## 6. Conclusions

This review highlights the significant potential of nanocarriers to address the persistent challenges associated with cART in mitigating HAND. Physiological challenges that make HAND hard to treat include chronic neuroinflammation, neurotoxicity caused by viral proteins, excitotoxicity, and the presence of the BBB. Although cART has substantially improved the life expectancy of people living with HIV, its effectiveness in preventing or reversing neurological complications remains limited. However, lifestyle modifications, socio-behavioral interventions, cognitive training, and adherence to available treatments can improve brain health and patient survival. The current long-term treatment regimen and continuous viral load monitoring are critical for the suppression of viral load and reducing neuroinflammation. Additionally, physical activity, a balanced diet, mental health counseling, and cognitive training could also improve overall mental health, along with reduced neuroinflammation and viral replication.

The application of nanocarrier-based drug delivery platforms offers a promising strategy to overcome these limitations by enabling improved targeting across the BBB, enhancing drug stability, and improving bioavailability. The developed formulations could be evaluated for therapeutic efficacy in different animal models as a preclinical assessment. These animals can be used to study the mechanisms underlying HAND progression, identify new biomarkers, mimic human disease pathology, and test new therapeutic tactics. Moreover, nanotechnology may improve patient compliance by reducing dosing frequency. Advanced polymeric nanocarriers could enhance antiretroviral penetration across the BBB and target viral reservoirs. Functionalization of these nanocarriers may enable cell-specific targeting and minimize toxicity. Additionally, nanosensors and gene-editing tools could be helpful in early diagnosis and precise eradication of latent virus in the CNS, respectively. Collectively, these advantages suggest that nanocarrier platforms could significantly advance the prevention and management of HAND using current cART regimens.

## Figures and Tables

**Table 1:** Lipid nanocarrier-based formulations for antiviral drug delivery to the brain against HAND.

Nanocarrier Type	Drug	Inference	Reference
Liposomes	3’-azido-3’-deoxythymidine	Localized accumulation of the drug with reduced toxicity and improved bioavailability	[38]
Liposomes	Nevirapine	Higher drug loading with stability at physiological pH	[39]
Liposomes	Tenofovir, Nelfinavir, methamphetamine	Enhanced bioavailability, permeability, and therapeutic efficacy	[40]
Liposomes	Cannabidiol	Reduced p24 expression in infected microglia	[41]
SLNs	ApoE	Higher permeability across the hCMEC/D3 monolayer	[43]
SLNs	Atazanavir	Reduced toxicity and greater cellular accumulation in the endothelial cell monolayer	[44]
SLNs	Stavudine, delaviridine, saquinavir	Crossed the BBB efficiently and demonstrated higher bioavailability	[45]
SLNs	Ritonavir	Significant inhibition of in vitro HIV-1 infection	[46]
NLCs	Atazanavir	Higher brain bioavailability	[49]
NLCs	Etravirine	Reduced HIV-1 viral load in anatomical reservoirs	[50]
NLCs	Tenofovir	Improved pharmacokinetics of the drug	[51]
NLCs	Etravirine	Improved biodistribution in the brain	[54]
NLCs	Etravirine	Improved pharmacokinetics	[55]

**Table 2:** Synthetic nanocarriers for drug delivery to the brain against HAND.

Nanocarrier	Drug	Inference	Reference
PBCA nanoparticles	Zidovudine, Lamivudine	Increased permeability across the BBB and improved bioavailability	[53]
PLA-PEG nanoparticles	Zidovudine	Enhanced retention time in the systemic circulation	[57]
PLGA nanoparticles	Ritonavir, Lopinavir, Efavirenz	Improved bioavailability in the brain and sustained in vitro anti-viral efficacy	[58]
PLGA nanoparticles	Darunavir	Improved permeability across the BBB and inhibited the metalloproteinase 9	[59]
PLA nanoparticles	Ritonavir	Improved bioavailability of the drug	[46]
Carbosilane-dendrimer	siRNA	Downregulation of GAPDH and restriction of HIV replication	[60]
Magnetic nanoparticles-exosomes	T20 peptide	Neuroprotection	[61]
Nanocrystal	Rilpivirine, Cabotegravir	Improved bioavailability	[62]
Dendrimer	Lamivudine, efavirenz	Reduced retroviral activity	[63]

**Table 3:** Apparent, construct, predictive, and convergent validity profiles of different HIV-animal models.

Animal Model	Validity				Reference
	Apparent	Construct	Predictive	Convergent	
*Humanized mice model*	Moderate	Moderate–High	Moderate	Moderate–High	[90]
*HIV-transgenic mice*	Moderate–High	Low–Moderate	Low–Moderate	Moderate	[91]
*Chimeric virus infection model*	Moderate	Moderate	Moderate	Moderate–High	[78]
*Simian-human immunodeficiency virus*	High	High	Moderate–High	High	[92]

## Data Availability

No new data were created or analyzed in this study.

## Abbreviations

HAND	HIV-associated neurocognitive disorders
BBB	blood–brain barrier
CNS	central nervous system
cART	combination antiretroviral therapy
SLN	solid lipid nanoparticles
NLC	nanostructured lipid carriers
PBCA	poly(butyl cyanoacrylate)
PLA	polylactic acid
PLGA	poly(lactic-co-glycolic acid)

## References

[1] United Nations (2024). Regional Chapters. UNAIDS Global AIDS Update 2024.

[2] Zenebe Y., Necho M., Yimam W., Akele B. (2022). Worldwide Occurrence of HIV-Associated Neurocognitive Disorders and Its Associated Factors: A Systematic Review and Meta-Analysis. Front. Psychiatry.

[3] Wang Y., Liu M., Lu Q., Farrell M., Lappin J.M., Shi J., Lu L., Bao Y. (2020). Global Prevalence and Burden of HIV-Associated Neurocognitive Disorder. Neurology.

[4] Jessen F., Broich K. (2025). Dementia: Changes from ICD-10 to ICD-11. Nervenarzt.

[5] Saylor D., Dickens A.M., Sacktor N., Haughey N., Slusher B., Pletnikov M., Mankowski J.L., Brown A., Volsky D.J., McArthur J.C. (2016). HIV-Associated Neurocognitive Disorder—Pathogenesis and Prospects for Treatment. Nat. Rev. Neurol..

[6] Pulliam L. (2024). Evolving Biomarkers for HIV-Associated Neurocognitive Disorders (HAND). HIV-Associated Neurocognitive Disorders.

[7] Maki P.M., Martin-Thormeyer E. (2009). HIV, Cognition and Women. Neuropsychol. Rev..

[8] Kaul M., Zheng J., Okamoto S., Gendelman H.E., Lipton S.A. (2005). HIV-1 Infection and AIDS: Consequences for the Central Nervous System. Cell Death Differ..

[9] Nguyen H.D., Kim W.-K. (2025). New Genetic Insights into HIV-Associated Neurocognitive Disorder and Alzheimer’s Disease. Genes Dis..

[10] Brew B.J., Pemberton L., Cunningham P., Law M.G. (1997). Levels of Human Immunodeficiency Virus Type 1 RNA in Cerebrospinal Fluid Correlate with AIDS Dementia Stage. J. Infect. Dis..

[11] McArthur J.C., McClernon D.R., Cronin M.F., Nance-Sproson T.E., Saah A.J., St Clair M., Lanier E.R. (1997). Relationship between Human Immunodeficiency Virus—Associated Dementia and Viral Load in Cerebrospinal Fluid and Brain. Ann. Neurol..

[12] Fujimura R.K., Shapshak P., Goodkin K., Petito C.K. (1998). Re: Distribution of Brain HIV Load in AIDS, Wiley CA et al., Brain Pathology 8: 277–284, 1998. Brain Pathol..

[13] Saxena S.K., Sharma D., Kumar S., Puri B. (2023). Understanding HIV-Associated Neurocognitive and Neurodegenerative Disorders (NeuroAIDS): Enroute to Achieve the 95-95-95 Target and Sustainable Development Goal for HIV/AIDS Response. Virusdisease.

[14] Kaul M., Garden G.A., Lipton S.A. (2001). Pathways to Neuronal Injury and Apoptosis in HIV-Associated Dementia. Nature.

[15] Everall I.P., Heaton R.K., Marcotte T.D., Ellis R.J., McCutchan J.A., Atkinson J.H., Grant I., Mallory M., Masliah E. (1999). Cortical Synaptic Density Is Reduced in Mild to Moderate Human Immunodeficiency Virus Neurocognitive Disorder. Brain Pathol..

[16] Di Liberto G., Egervari K., Kreutzfeldt M., Schürch C.M., Hewer E., Wagner I., Du Pasquier R., Merkler D. (2022). Neurodegenerative Phagocytes Mediate Synaptic Stripping in Neuro-HIV. Brain.

[17] Ranki A., Nyberg M., Ovod V., Haltia M., Elovaara I., Raininko R., Haapasalo H., Krohn K. (1995). Abundant Expression of HIV Nef and Rev Proteins in Brain Astrocytes In Vivo Is Associated with Dementia. AIDS.

[18] Hudson L., Liu J., Nath A., Jones M., Raghavan R., Narayan O., Male D., Everall I. (2000). Detection of the Human Immunodeficiency Virus Regulatory Protein *Tat* in CNS Tissues. J. Neurovirol..

[19] Jones M.V., Bell J.E., Nath A. (2000). Immunolocalization of HIV Envelope Gp120 in HIV Encephalitis with Dementia. AIDS.

[20] Vignoli A.L., Martini I., Haglid K.H., Silvestroni L., Augusti-Tocco G., Biagioni S. (2000). Neuronal Glycolytic Pathway Impairment Induced by HIV Envenlope Glyocprotein Gp120. Mol. Cell. Biochem..

[21] Vesce S., Bezzi P., Rossi D., Meldolesi J., Volterra A. (1997). HIV-1 Gp120 Glycoprotein Affects the Astrocyte Control of Extracellular Glutamate by Both Inhibiting the Uptake and Stimulating the Release of the Amino Acid. FEBS Lett..

[22] Erdmann N.B., Whitney N.P., Zheng J. (2006). Potentiation of Excitotoxicity in HIV-1-Associated Dementia and the Significance of Glutaminase. Clin. Neurosci. Res..

[23] Murphy A.J., Kelschenbach J., He H., Chao W., Kim B.-H., Volsky D.J., Berman J.W. (2022). Buprenorphine Reverses Neurocognitive Impairment in EcoHIV Infected Mice: A Potential Therapy for HIV-NCI. Front. Immunol..

[24] Löscher W., Potschka H. (2005). Blood-Brain Barrier Active Efflux Transporters: ATP-Binding Cassette Gene Family. NeuroRX.

[25] Chan G.N.Y., Patel R., Cummins C.L., Bendayan R. (2013). Induction of P-Glycoprotein by Antiretroviral Drugs in Human Brain Microvessel Endothelial Cells. Antimicrob. Agents Chemother..

[26] Edagwa B., Zhou T., McMillan J., Liu X.-M., Gendelman H. (2014). Development of HIV Reservoir Targeted Long Acting Nanoformulated Antiretroviral Therapies. Curr. Med. Chem..

[27] Wallet C., De Rovere M., Van Assche J., Daouad F., De Wit S., Gautier V., Mallon P.W.G., Marcello A., Van Lint C., Rohr O. (2019). Microglial Cells: The Main HIV-1 Reservoir in the Brain. Front. Cell. Infect. Microbiol..

[28] Ricardo-Dukelow M., Kadiu I., Rozek W., Schlautman J., Persidsky Y., Ciborowski P., Kanmogne G.D., Gendelman H.E. (2007). HIV-1 Infected Monocyte-Derived Macrophages Affect the Human Brain Microvascular Endothelial Cell Proteome: New Insights into Blood–Brain Barrier Dysfunction for HIV-1-Associated Dementia. J. Neuroimmunol..

[29] Brack-Werner R. (1999). Astrocytes: HIV Cellular Reservoirs and Important Participants in Neuropathogenesis. AIDS.

[30] Osborne O., Peyravian N., Nair M., Daunert S., Toborek M. (2020). The Paradox of HIV Blood–Brain Barrier Penetrance and Antiretroviral Drug Delivery Deficiencies. Trends Neurosci..

[31] Upadhyay R.K. (2014). Drug Delivery Systems, CNS Protection, and the Blood Brain Barrier. BioMed Res. Int..

[32] Niazi S.K. (2023). Non-Invasive Drug Delivery across the Blood–Brain Barrier: A Prospective Analysis. Pharmaceutics.

[33] Armada-Moreira A., Gomes J.I., Pina C.C., Savchak O.K., Gonçalves-Ribeiro J., Rei N., Pinto S., Morais T.P., Martins R.S., Ribeiro F.F. (2020). Going the Extra (Synaptic) Mile: Excitotoxicity as the Road Toward Neurodegenerative Diseases. Front. Cell. Neurosci..

[34] Was H., Borkowska A., Bagues A., Tu L., Liu J.Y.H., Lu Z., Rudd J.A., Nurgali K., Abalo R. (2022). Mechanisms of Chemotherapy-Induced Neurotoxicity. Front. Pharmacol..

[35] Nair M., Jayant R.D., Kaushik A., Sagar V. (2016). Getting into the Brain: Potential of Nanotechnology in the Management of NeuroAIDS. Adv. Drug Deliv. Rev..

[36] Wu D., Chen Q., Chen X., Han F., Chen Z., Wang Y. (2023). The Blood–Brain Barrier: Structure, Regulation and Drug Delivery. Signal Transduct. Target. Ther..

[37] Dusserre N., Lessard C., Paquette N., Perron S., Poulin L., Tremblay M., Beauchamp D., Désormeaux A., Bergeron M.G. (1995). Encapsulation of Foscarnet in Liposomes Modifies Drug Intracellular Accumulation, In Vitro Anti-HIV-1 Activity, Tissue Distribution, and Pharmacokinetics. AIDS.

[38] Phillips N.C., Tsoukas C. (1991). Liposomal Encapsulation of Azt Results in Decreased Bone Marrow Toxicity and Activity against Murine Acquired Immunodeficiency Syndrome (MAIDS)-Induced Immunosuppression. Int. J. Immunopharmacol..

[39] Ramana L.N., Sethuraman S., Ranga U., Krishnan U.M. (2010). Development of a Liposomal Nanodelivery System for Nevirapine. J. Biomed. Sci..

[40] Jayant R.D., Tiwari S., Atluri V., Kaushik A., Tomitaka A., Yndart A., Colon-Perez L., Febo M., Nair M. (2018). Multifunctional Nanotherapeutics for the Treatment of NeuroAIDS in Drug Abusers. Sci. Rep..

[41] Yndart Arias A., Vashist A., Vadell K., Lakshmana M.K., Liuzzi J.P. (2025). Liposomal-Cannabidiol Nanoformulation to Suppress HIV Replication and Reduce Oxidative Stress in Infected Microglia. ACS Biomater. Sci. Eng..

[42] Kuo Y.-C., Shih-Huang C.-Y. (2014). Solid Lipid Nanoparticles with Surface Antibody for Targeting the Brain and Inhibiting Lymphatic Phagocytosis. J. Taiwan Inst. Chem. Eng..

[43] Neves A.R., Queiroz J.F., Weksler B., Romero I.A., Couraud P.-O., Reis S. (2015). Solid Lipid Nanoparticles as a Vehicle for Brain-Targeted Drug Delivery: Two New Strategies of Functionalization with Apolipoprotein E. Nanotechnology.

[44] Chattopadhyay N., Zastre J., Wong H.-L., Wu X.Y., Bendayan R. (2008). Solid Lipid Nanoparticles Enhance the Delivery of the HIV Protease Inhibitor, Atazanavir, by a Human Brain Endothelial Cell Line. Pharm. Res..

[45] Kuo Y., Su F. (2007). Transport of Stavudine, Delavirdine, and Saquinavir across the Blood–Brain Barrier by Polybutylcyanoacrylate, Methylmethacrylate-Sulfopropylmethacrylate, and Solid Lipid Nanoparticles. Int. J. Pharm..

[46] Javan F., Vatanara A., Azadmanesh K., Nabi-Meibodi M., Shakouri M. (2017). Encapsulation of Ritonavir in Solid Lipid Nanoparticles: In-Vitro Anti-HIV-1 Activity Using Lentiviral Particles. J. Pharm. Pharmacol..

[47] Prasad M., Lambe U.P., Brar B., Shah I., Manimegalai J., Ranjan K., Rao R., Kumar S., Mahant S., Khurana S.K. (2018). Nanotherapeutics: An Insight into Healthcare and Multi-Dimensional Applications in Medical Sector of the Modern World. Biomed. Pharmacother..

[48] Aggarwal N., Sachin, Nabi B., Aggarwal S., Baboota S., Ali J. (2021). Nano-Based Drug Delivery System: A Smart Alternative towards Eradication of Viral Sanctuaries in Management of NeuroAIDS. Drug Deliv. Transl. Res..

[49] Khan S.A., Rehman S., Nabi B., Iqubal A., Nehal N., Fahmy U.A., Kotta S., Baboota S., Md S., Ali J. (2020). Boosting the Brain Delivery of Atazanavir through Nanostructured Lipid Carrier-Based Approach for Mitigating NeuroAIDS. Pharmaceutics.

[50] Rojekar S., Fotooh Abadi L., Pai R., Mahajan K., Kulkarni S., Vavia P.R. (2021). Multi-Organ Targeting of HIV-1 Viral Reservoirs with Etravirine Loaded Nanostructured Lipid Carrier: An In-Vivo Proof of Concept. Eur. J. Pharm. Sci..

[51] Sarma A., Das M.K. (2019). Formulation by Design (FbD) Approach to Develop Tenofovir Disoproxil Fumarate Loaded Nanostructured Lipid Carriers (NLCs) for the Aptness of Nose to Brain Delivery. J. Drug Deliv. Ther..

[52] Kumar S., Maurya V.K., Dandu H.R., Bhatt M.L.B., Saxena S.K. (2018). Global Perspective of Novel Therapeutic Strategies for the Management of NeuroAIDS. Biomol. Concepts.

[53] Kuo Y.-C., Chen H.-H. (2006). Effect of Nanoparticulate Polybutylcyanoacrylate and Methylmethacrylate–Sulfopropylmethacrylate on the Permeability of Zidovudine and Lamivudine across the In Vitro Blood–Brain Barrier. Int. J. Pharm..

[54] Rojekar S., Abadi L.F., Pai R., Prajapati M.K., Kulkarni S., Vavia P.R. (2022). Mannose-Anchored Nano-Selenium Loaded Nanostructured Lipid Carriers of Etravirine for Delivery to HIV Reservoirs. AAPS PharmSciTech.

[55] Rojekar S., Pai R., Abadi L.F., Mahajan K., Prajapati M.K., Kulkarni S., Vavia P. (2021). Dual Loaded Nanostructured Lipid Carrier of Nano-Selenium and Etravirine as a Potential Anti-HIV Therapy. Int. J. Pharm..

[56] Sagar V., Pilakka-Kanthikeel S., Pottathil R., Saxena S.K., Nair M. (2014). Towards Nanomedicines for NeuroAIDS. Rev. Med. Virol..

[57] Mainardes R.M., Gremião M.P.D., Brunetti I.L., da Fonseca L.M., Khalil N.M. (2009). Zidovudine-Loaded PLA and PLA–PEG Blend Nanoparticles: Influence of Polymer Type on Phagocytic Uptake by Polymorphonuclear Cells. J. Pharm. Sci..

[58] Destache C.J., Belgum T., Goede M., Shibata A., Belshan M.A. (2010). Antiretroviral Release from Poly(DL-Lactide-Co-Glycolide) Nanoparticles in Mice. J. Antimicrob. Chemother..

[59] Latronico T., Rizzi F., Panniello A., Laquintana V., Arduino I., Denora N., Fanizza E., Milella S., Mastroianni C.M., Striccoli M. (2021). Luminescent PLGA Nanoparticles for Delivery of Darunavir to the Brain and Inhibition of Matrix Metalloproteinase-9, a Relevant Therapeutic Target of HIV-Associated Neurological Disorders. ACS Chem. Neurosci..

[60] Jiménez J.L., Clemente M.I., Weber N.D., Sanchez J., Ortega P., de la Mata F.J., Gómez R., García D., López-Fernández L.A., Muñoz-Fernández M.Á. (2010). Carbosilane Dendrimers to Transfect Human Astrocytes with Small Interfering RNA Targeting Human Immunodeficiency Virus. BioDrugs.

[61] Caobi A., Andre M., Miles J., Tomitaka A., Nikkhah-Moshaie R., Hernandez A., Nair M., Raymond A.D. (2020). Magnetic Nanoparticle and Exosomal Therapeutic (M-NEXT) Effects on HIV-Associated Neurotoxicity. Crit. Rev. Biomed. Eng..

[62] Abbate M.T.A., Ramöller I.K., Sabri A.H., Paredes A.J., Hutton A.J., McKenna P.E., Peng K., Hollett J.A., McCarthy H.O., Donnelly R.F. (2023). Formulation of Antiretroviral Nanocrystals and Development into a Microneedle Delivery System for Potential Treatment of HIV-Associated Neurocognitive Disorder (HAND). Int. J. Pharm..

[63] Pargoo E.M., Aghasadeghi M.R., Parivar K., Nikbin M., Rahimi P., Ardestani M.S. (2021). Lamivudine-conjugated and Efavirenz-loaded G2 Dendrimers: Novel Anti-retroviral Nano Drug Delivery Systems. IET Nanobiotechnol..

[64] Rao K.S., Reddy M.K., Horning J.L., Labhasetwar V. (2008). TAT-Conjugated Nanoparticles for the CNS Delivery of Anti-HIV Drugs. Biomaterials.

[65] Omeragic A., Kayode O., Hoque M.T., Bendayan R. (2020). Potential Pharmacological Approaches for the Treatment of HIV-1 Associated Neurocognitive Disorders. Fluids Barriers CNS.

[66] Blunt T., Gell D., Fox M., Taccioli G.E., Lehmann A.R., Jackson S.P., Jeggo P.A. (1996). Identification of a Nonsense Mutation in the Carboxyl-Terminal Region of DNA-Dependent Protein Kinase Catalytic Subunit in the Scid Mouse. Proc. Natl. Acad. Sci. USA.

[67] Xiong H., Zeng Y.C., Zheng J., Thylin M., Gendelman H.E. (1999). Soluble HIV-1 Infected Macrophage Secretory Products Mediate Blockade of Long-Term Potentiation: A Mechanism for Cognitive Dysfunction in HIV-1-Associated Dementia. J. Neurovirol..

[68] Poluektova L., Gorantla S., Faraci J., Birusingh K., Dou H., Gendelman H.E. (2004). Neuroregulatory Events Follow Adaptive Immune-Mediated Elimination of HIV-1-Infected Macrophages: Studies in a Murine Model of Viral Encephalitis. J. Immunol..

[69] Poluektova L.Y., Munn D.H., Persidsky Y., Gendelman H.E. (2002). Generation of Cytotoxic T Cells Against Virus-Infected Human Brain Macrophages in a Murine Model of HIV-1 Encephalitis. J. Immunol..

[70] Wege A.K., Melkus M.W., Denton P.W., Estes J.D., Garcia J.V. (2008). Functional and Phenotypic Characterization of the Humanized BLT Mouse Model. Humanized Mice.

[71] Asahchop E.L., Meziane O., Mamik M.K., Chan W.F., Branton W.G., Resch L., Gill M.J., Haddad E., Guimond J.V., Wainberg M.A. (2017). Reduced Antiretroviral Drug Efficacy and Concentration in HIV-Infected Microglia Contributes to Viral Persistence in Brain. Retrovirology.

[72] Toggas S.M., Masliah E., Rockenstein E.M., Rail G.F., Abraham C.R., Mucke L. (1994). Central Nervous System Damage Produced by Expression of the HIV-1 Coat Protein Gpl20 in Transgenic Mice. Nature.

[73] Garden G.A., Budd S.L., Tsai E., Hanson L., Kaul M., D’Emilia D.M., Friedlander R.M., Yuan J., Masliah E., Lipton S.A. (2002). Caspase Cascades in Human Immunodeficiency Virus-Associated Neurodegeneration. J. Neurosci..

[74] Kim B.O., Liu Y., Ruan Y., Xu Z.C., Schantz L., He J.J. (2003). Neuropathologies in Transgenic Mice Expressing Human Immunodeficiency Virus Type 1 Tat Protein under the Regulation of the Astrocyte-Specific Glial Fibrillary Acidic Protein Promoter and Doxycycline. Am. J. Pathol..

[75] Acharjee S., Noorbakhsh F., Stemkowski P.L., Olechowski C., Cohen E.A., Ballanyi K., Kerr B., Pardo C., Smith P.A., Power C. (2010). HIV-1 Viral Protein R Causes Peripheral Nervous System Injury Associated with In Vivo Neuropathic Pain. FASEB J..

[76] Valle-Tenney R., Opazo T., Cancino J., Goff S.P., Arriagada G. (2016). Dynein Regulators Are Important for Ecotropic Murine Leukemia Virus Infection. J. Virol..

[77] Ramsdale E.E., Kingsman S.M., Kingsman A.J. (1996). The “Putative” Leucine Zipper Region of Murine Leukemia Virus Transmembrane Protein (P15e) Is Essential for Viral Infectivity. Virology.

[78] Potash M.J., Chao W., Bentsman G., Paris N., Saini M., Nitkiewicz J., Belem P., Sharer L., Brooks A.I., Volsky D.J. (2005). A Mouse Model for Study of Systemic HIV-1 Infection, Antiviral Immune Responses, and Neuroinvasiveness. Proc. Natl. Acad. Sci. USA.

[79] Adachi A., Gendelman H.E., Koenig S., Folks T., Willey R., Rabson A., Martin M.A. (1986). Production of Acquired Immunodeficiency Syndrome-Associated Retrovirus in Human and Nonhuman Cells Transfected with an Infectious Molecular Clone. J. Virol..

[80] Spire B., Sire J., Zachar V., Rey F., Barré-Sinoussi F., Galibert F., Hampe A., Chermann J.-C. (1989). Nucleotide Sequence of HIV1-NDK: A Highly Cytopathic Strain of the Human Immunodeficiency Virus. Gene.

[81] Anderson D.J., Politch J.A. (2014). Remarks on the Article of Hadas et al.: Transmission of Chimeric HIV by Mating in Conventional Mice: Prevention by Pre-Exposure Antiretroviral Therapy and Reduced Susceptibility during Estrus. Dis. Models Mech..

[82] Hadas E., Borjabad A., Chao W., Saini M., Ichiyama K., Potash M.J., Volsky D.J. (2007). Testing Antiretroviral Drug Efficacy in Conventional Mice Infected with Chimeric HIV-1. AIDS.

[83] Nedelcovych M.T., Tenora L., Kim B.-H., Kelschenbach J., Chao W., Hadas E., Jančařík A., Prchalová E., Zimmermann S.C., Dash R.P. (2017). N-(Pivaloyloxy)Alkoxy-Carbonyl Prodrugs of the Glutamine Antagonist 6-Diazo-5-Oxo-l-Norleucine (DON) as a Potential Treatment for HIV Associated Neurocognitive Disorders. J. Med. Chem..

[84] Skowronska M., McDonald M., Velichkovska M., Leda A.R., Park M., Toborek M. (2018). Methamphetamine Increases HIV Infectivity in Neural Progenitor Cells. J. Biol. Chem..

[85] Tomusange K., Wijesundara D., Gummow J., Garrod T., Li Y., Gray L., Churchill M., Grubor-Bauk B., Gowans E.J. (2016). A HIV-Tat/C4-Binding Protein Chimera Encoded by a DNA Vaccine Is Highly Immunogenic and Contains Acute EcoHIV Infection in Mice. Sci. Rep..

[86] Sindberg G.M., Sharma U., Banerjee S., Anand V., Dutta R., Gu C.-J., Volsky D.J., Roy S. (2014). An Infectious Murine Model for Studying the Systemic Effects of Opioids on Early HIV Pathogenesis in the Gut. J. Neuroimmune Pharmacol..

[87] Kumar N., Chahroudi A., Silvestri G. (2016). Animal Models to Achieve an HIV Cure. Curr. Opin. HIV AIDS.

[88] Parker R.A., Regan M.M., Reimann K.A. (2001). Variability of Viral Load in Plasma of Rhesus Monkeys Inoculated with Simian Immunodeficiency Virus or Simian-Human Immunodeficiency Virus: Implications for Using Nonhuman Primate AIDS Models to Test Vaccines and Therapeutics. J. Virol..

[89] Garcia-Tellez T., Huot N., Ploquin M.J., Rascle P., Jacquelin B., Müller-Trutwin M. (2016). Non-Human Primates in HIV Research: Achievements, Limits and Alternatives. Infect. Genet. Evol..

[90] Honeycutt J.B., Garcia J.V. (2017). Humanized Mice: Models for Evaluating NeuroHIV and Cure Strategies. J. Neurovirol..

[91] Thaney V.E., Sanchez A.B., Fields J.A., Minassian A., Young J.W., Maung R., Kaul M. (2017). Transgenic Mice Expressing HIV-1 Envelope Protein Gp120 in the Brain as an Animal Model in NeuroAIDS Research. J. Neurovirol..

[92] Moretti S., Virtuoso S., Sernicola L., Farcomeni S., Maggiorella M.T., Borsetti A. (2021). Advances in SIV/SHIV Non-Human Primate Models of NeuroAIDS. Pathogens.

[93] Bauer A.M., Ziani W., Lindemuth E., Kuri-Cervantes L., Li H., Lee F.-H., Watkins M., Ding W., Xu H., Veazey R. (2020). Novel Transmitted/Founder Simian-Human Immunodeficiency Viruses for Human Immunodeficiency Virus Latency and Cure Research. J. Virol..

[94] Saloner R., Cysique L.A. (2017). HIV-Associated Neurocognitive Disorders: A Global Perspective. J. Int. Neuropsychol. Soc..

[95] Kamminga J., Cysique L.A., Lu G., Batchelor J., Brew B.J. (2013). Validity of Cognitive Screens for HIV-Associated Neurocognitive Disorder: A Systematic Review and an Informed Screen Selection Guide. Curr. HIV/AIDS Rep..

[96] Maki P.M., Rubin L.H., Valcour V., Martin E., Crystal H., Young M., Weber K.M., Manly J., Richardson J., Alden C. (2015). Cognitive Function in Women with HIV. Neurology.

